# The carboxypeptidase B and carbonic anhydrase genes play a reproductive regulatory role during multiple matings in *Ophraella communa*

**DOI:** 10.3389/fmolb.2023.1095645

**Published:** 2023-05-17

**Authors:** Guangmei Chen, Xuyuan Gao, Yan Zhang, Chao Ma, Weihua Ma, Zhongshi Zhou

**Affiliations:** ^1^ State Key Laboratory for Biology of Plant Diseases and Insect Pests, Institute of Plant Protection, Chinese Academy of Agricultural Sciences, Beijing, China; ^2^ National Nanfan Research Institute, Chinese Academy of Agricultural Sciences, Sanya, China; ^3^ Guangxi Key Laboratory for Biology of Crop Diseases and Insect Pests, Institute of Plant Protection, Guangxi Academy of Agricultural Sciences, Nanning, China; ^4^ College of Plant Science and Technology, Huazhong Agricultural University, Wuhan, China

**Keywords:** multiple mating, seminal fluid protein genes, female reproductive tract, reproduction, *Ophraella communa*

## Abstract

Seminal fluid proteins (SFPs) are key factors in sexual reproduction and are transferred to females during mating with sperm. SFPs have a nutritional value because they protect and activate sperm storage and release to optimize fecundity. Multiple matings promote ovipositioning in several insect species. Therefore, insects may obtain more SFP through multiple matings to maximize reproduction, but this process has not yet been clearly confirmed. Here, the relationship between multiple matings and the SFPs in *Ophraella communa* (Coleoptera: Chrysomelidae), a biological control agent of the common ragweed *Ambrosia artemisiifolia* (Asterales: Asteraceae), was studied. Multiple matings significantly increased female fecundity and ovary egg deposition. Carboxypeptidase B (*OcCpb*) and carbonic anhydrase (*OcCa*) genes were identified as putative SFP genes in *O. communa* and they showed strong male-biased expression. Additionally, *OcCpb* and *OcCa* expression was upregulated in the bursa copulatrix of mating females compared to that in virgin females, but their expression gradually declined after copulation. Furthermore, *OcCpb* and *OcCa* knockdown in males led to a decrease in insect fecundity compared to that in the control. The reproductive tract of females mated with dsRNA-treated males was dissected and observed and, notably, the ovaries produced significantly fewer eggs. These data suggest that *OcCpb* and *OcCa* play regulatory roles during multiple matings in *O. communa*.

## Introduction

Multiple matings are a reproductive process that affects the expansion of insect populations. They are classified as monandrous or polyandrous (Newcomer et al., 1999). However, females may be more willing to re-mate with familiar males to avoid any physical damage caused by the genitals during mating (Caesar and Forsman, 2009). Moreover, monandrous multiple matings can provide females with additional benefits (Wang et al., 2018), such as increased lifetime fecundity (Alcock et al., 1978; Ward and Landolt, 1995) and the nutrients from male ejaculates (Arnqvist and Nilsson, 2000). Therefore, most females may obtain physiological or genetic benefits from multiple mating (Jennions and Petrie, 2007). Furthermore, if females receive insufficient ejaculate from one mating, they may mate multiple times to ensure that all their eggs are fertilized (Eberhard, 1996; Simmons, 2019).

Females initiate ovipositioning behavior after mating in sexually reproducing taxa. Seminal fluid proteins (SFPs), which are also known as male accessory gland proteins (Acps), are key factors in sexual reproduction. Insect SFPs are produced in the male reproductive tract (MRT) secretory tissues (testes, seminal vesicles, accessory glands, *etc.*) (Avila et al., 2011). They are composed of many substances, including polypeptides, lectins, proteases, protease inhibitors, protective proteins similar to antioxidants, and substances that do not encode proteins (Poiani, 2006; Pondeville et al., 2008). SFPs are transferred with sperm to females during mating, and they induce physiological and behavioral changes in females (Neubaum and Wolfner, 1999; Koene et al., 2010), such as decreasing receptivity to re-mating, altering feeding behaviors, and increasing ovulation and egg-laying rate (Avila et al., 2011; Immarigeon et al., 2021). Additionally, the process of coordinates gametes for fertilization are triggered by SFPs in *Drosophila melanogaster*. (Mann et al., 1982; Bloch Qazi et al., 2003).

Developments in proteomic and RNA interference techniques have advanced the identification and functional analysis of SFPs in many insect species (Avila et al., 2011). SFPs undergo molecular interactions that cause behavioral changes in females post mating and have been shown to be the main functional proteins affecting insect reproduction (Lung and Wolfner, 2001; Mueller et al., 2008). For example, seminal fluid signals encourage females to allocate resources to the ova, resulting in greater egg production in *Ephestia kuehniella* (Xu and Wang, 2011). RNAi technology has benefited insect system studies. These techniques have shown that double-stranded RNA (dsRNA) or siRNA injections into adult or juvenile insects can successfully knockdown gene transcripts in a diverse array of taxa (Marshall et al., 2009). Furthermore, the downregulation of 15 tested SFP genes substantially decreased defensive first male paternity success in *D. melanogaster* (Patlar and Civetta, 2022). In addition, knockout of the SFP gene, *BmSfp62,* led to male sterility in *Bombyx mori* (Xu et al., 2022), and knockdown of the gene encoding for a protein similar to angiotensin-converting enzyme in *Tribolium castaneum* males reduced female fecundity after mating (Xu et al., 2013). Thus, it is possible to assess the function of individual proteins and their roles in mediating reproductive physiology using RNAi technology.

*Ophraella communa* Lesage (Coleoptera: Chrysomelidae) is a specific and effective natural enemy of the invasive common ragweed *Ambrosia artemisiifolia* L. (Asterales: Asteraceae) (Zhou et al., 2011). This specialist herbivore is native to North America (Futuyma and McCafferty, 1990; Hu and Meng, 2007) and was first discovered in Nanjing, Jiangsu Province, China, in 2001 (Meng and Li, 2005). It has been widely used as a biological control agent of *A. artemisiifolia* in Canada and China (Mason and Huber, 2002; Zhou et al., 2009; 2010; Guo et al., 2011). *Ophraella communa* adults mate several times throughout the day and during their lifespan (Meng and Li, 2005; Zheng et al., 2011), and the number of copulation events is positively associated with insect fitness parameters (Zhou et al., 2015). Beetle mating behavior is visible, which means that it can used as a feasible parameter for mating regime studies.

The ejaculate substance stored by *T. castaneum* females increased by 33% in doubly mated females compared to that in singly mated females, indicating that the spermatheca was filled to only two-thirds of its capacity following insemination by the first male (Lewis and Jutkiewicz, 1998). Multiple matings by *O. communa* may have a cumulative effect that is similar to that in *T. castaneum* and involves SFPs transferred by mating. The high reproductive ability of the beetle is considered to be not only related to the physiological basis of the females but also to the regulation of the SFPs supplied by the males. However, the mechanism controlling male-mediated reproduction regulation remains unclear, especially whether insects maximize reproduction by obtaining more seminal proteins through multiple matings. Thus, we sampled mating and non-mating bursa copulatrix of females, and used transcriptomic and proteomic approaches to construct an *O. Communa* transferable SFPs database. In previous study, Gao et al. successfully identified a subset of transcriptional profiles of the testes and accessory glands of male *O. communa*. Following screening of this database, two SFP genes whose expression levels were modified in the bursa copulatrix were obtained—namely, carboxypeptidase B (EC 3.4.17.2, Cpb) and carbonic anhydrase (EC 4.2.1.1, Ca) genes.

Cpb is a carboxypeptidase that contains the peptidase_M14 domain and requires divalent metal ions such as Zn^2+^, for the specific hydrolysis of arginine and lysine residues at the C-terminus. It is a crucial enzyme in the digestive tract of insects and contributes to insect metamorphosis, development, and resistance (Barrett et al., 2012). Furthermore, Cpb activity has been reported in the male reproductive system of *Bombyx mori* (Aigaki et al., 1988)*.* Ca is a metalloenzyme that combines Zn^2+^ with its active centers. It was first discovered in human red blood cells (Meldrum and Roughton, 1933), and was subsequently reported in algae, fungi, and bacteria (Smith et al., 1999). Ca reversibly catalyzes the reaction between CO_2_ and HCO_3_^−^, and this reaction is involved in various physiological functions in organisms, enabling them to maintain their physiological activities (Mirjafari et al., 2007). A novel pH/HCO_3_^−^ dependent regulatory mechanism mediated by Ca is reported to be involved in the motility control in flatfish sperm (Inaba et al., 2003).

We hypothesized that SFP genes play a reproductive regulatory role in multiple matings of *O. communa* adults. To test this hypothesis, we created different mating regimes to explore the functions of *OcCpb* and *OcCa* in multiple matings between beetle adults. Technical methods, such as gene cloning, quantitative real-time PCR (qPCR), and RNAi were used in this study.

## Materials and methods

### *Ophraella communa* maintenance

*Ophraella communa* were collected from Laibin City, Guangxi Zhuang Autonomous Region, China, and reared on the common ragweed, *A. artemisiifolia*, in cages (40 × 60 cm) at 26°C ± 2°C and 70% ± 5% relative humidity, and under 14 h daylight. First, mature pupae were removed from the common ragweed leaves, and the first emerging male and female adults were regularly separated under a microscope at 9:00 a.m., 2:00 p.m., and 7:00 p.m. To avoid the errors caused by the emergence time interval, the adults that emerged at 2:00 p.m. and 7:00 p.m. were used for the experiment, and the remaining beetles were used for population expansion.

### Mating

Virgin females and males aged 5 days were randomly paired in a Petri dish (diameter = 6 cm) and their mating behavior was continuously observed from 8:00 a.m. to 10:00 p.m. The pairs (spouses) did not change during the study period. The mated females were reared alone in Petri dishes immediately after the mating treatment. There were five female mating regime treatments: mating once (M1), twice (M2), three (M3), and four (M4) times, and non-mating (NM) females, which were used as controls. Each treatment contained at least 30 replicates.

### Female reproductive tract observation

The female reproductive tract (FRT, Supplementary Figure S1) was dissected from virgin and mating-treatment females. Then, its structure and ovarian development were observed and ovarian morphology was recorded using a stereo fluorescence microscope (SZX 16, Olympus, Tokyo, Japan). There were 3–5 replicates. Following copulation, males generally transfer spermatophores to the female bursa copulatrix.

### Tissue dissection

The FRT (including ovary, median oviduct, and bursa copulatrix) and MRT (including testes, seminal vesicles, and accessory glands) were dissected from virgin adults aged 4 days in 1× phosphate buffered saline (PBS; pH = 7.4). The male head, thorax, gut, and fat were similarly dissected. Tissue samples were collected from six adults. The female bursa copulatrix was dissected within 2 min of mating. Each tissue was washed in PBS to remove any hemolymph and immediately frozen at −80°C.

### RNA extraction, cDNA synthesis, and gene cloning

Total RNA was isolated using TRIzol reagent (Invitrogen, Carlsbad, CA, United States) according to the manufacturer’s instructions. RNA purity and integrity were determined using spectrophotometry with a NanoDrop TM 1000 (Thermo Fisher Scientific, Waltham, MA, United States) and 1% agarose gel electrophoresis, respectively. Then, cDNA synthesis and gene cloning were performed as previously described (Ma et al., 2020; Tian et al., 2020). All the primers used in our study were designed using Primer Premier 5 (PREMIER Biosoft International, Palo Alto, CA, United States) and are listed in Supplementary Table S1.

### Sequence analysis

The molecular weights and isoelectric points of the amino acid sequences were calculated using ExPASy SERVER (http://www.expasy.ch/cgi-bin/pi_tool). The conserved sites were predicted using the InterProScan database (http://www.ebi.ac.uk/interpro/search/sequence/) and homologs of *Cpb* and *Ca* in coleopteran species were obtained from the NCBI database. They were aligned using ClustalX2 software and the GENEDOC program.

### qPCR analysis

The relative mRNA levels for *OcCpb* and *OcCa* in different tissues and developmental stages, and after injection of the dsRNA were determined using qPCR. The qPCR was performed using Hieff^®^ qPCR SYBR Green Master Mix (Low Rox Plus; Shanghai Yisheng Biotechnology Co., Ltd., China) and the following cycling program: initial incubation at 94°C for 5 min, followed by 40 cycles of 94°C for 30 s, and then 60°C for 34 s. The melting curve conditions were 95°C for 1 min, 60°C for 30 s, 95°C for 1 min, and 60°C for 30 s over one cycle. The quantitative mRNA measurements were performed in triplicate and normalized to the reference *O. communa* ribosomal protein L4 (*RPL4*) mRNA. The primers were synthesized at Shanghai Shenggong Biological Engineering Technology Service Co., Ltd., China and standard curves were obtained using a 2-fold serial dilution of the pooled cDNA.

### Double-stranded RNA synthesis and microinjection

The primers used to amplify ds*OcCpb*, ds*OcCa*, and the enhanced green fluorescent protein (ds*EGFP*, control treatment) are listed in Supplementary Table S1. After PCR amplification, the targeted fragment was used to synthesize dsRNA using a MEGAscript RNAi kit (Ambion Inc., Austin, TX, United States) according to the manufacturer’s instructions. The synthesized dsRNA was diluted to 10 μg μL^–1^ and stored at −20°C until needed.

Newly emerged *O. communa* (<6 h) males were microinjected with RNAi to explore the function of SFP. An agarose plate, which had been placed on an ice tray, was used to immobilize the insects. Then, dsRNA aliquots (0.1 μL) were injected into the abdomen of each *O. communa* male using a PLI-100 Pico-Injector (Harvard Apparatus, Holliston, MA, United States) and manipulated by an MP-255 micromanipulator (Sutter, Novato, CA, United States) under a microscope. The males were paired with virgin females of the same age at 3 days after injection. The females were separated immediately after mating once and were then fed in Petri dishes with fresh leaves of *A. artemisiifolia*. No less than 30 individuals were used for the oviposition assay and female fecundity was recorded daily from 1 to 15 days at 3:00 p.m.

### Statistical analysis

Statistical analyses were performed using SAS 9.4 (SAS Institute Inc., Cary, NC, United States). The qPCR data were analyzed using the 2^−ΔΔCT^ method, and the relative expression of SFP genes and fecundity were analyzed using one-way ANOVA and an LSD test at a significance level of *p* < 0.05.

## Results

### Effect of multiple matings on fecundity and FRT

**FIGURE 1:**
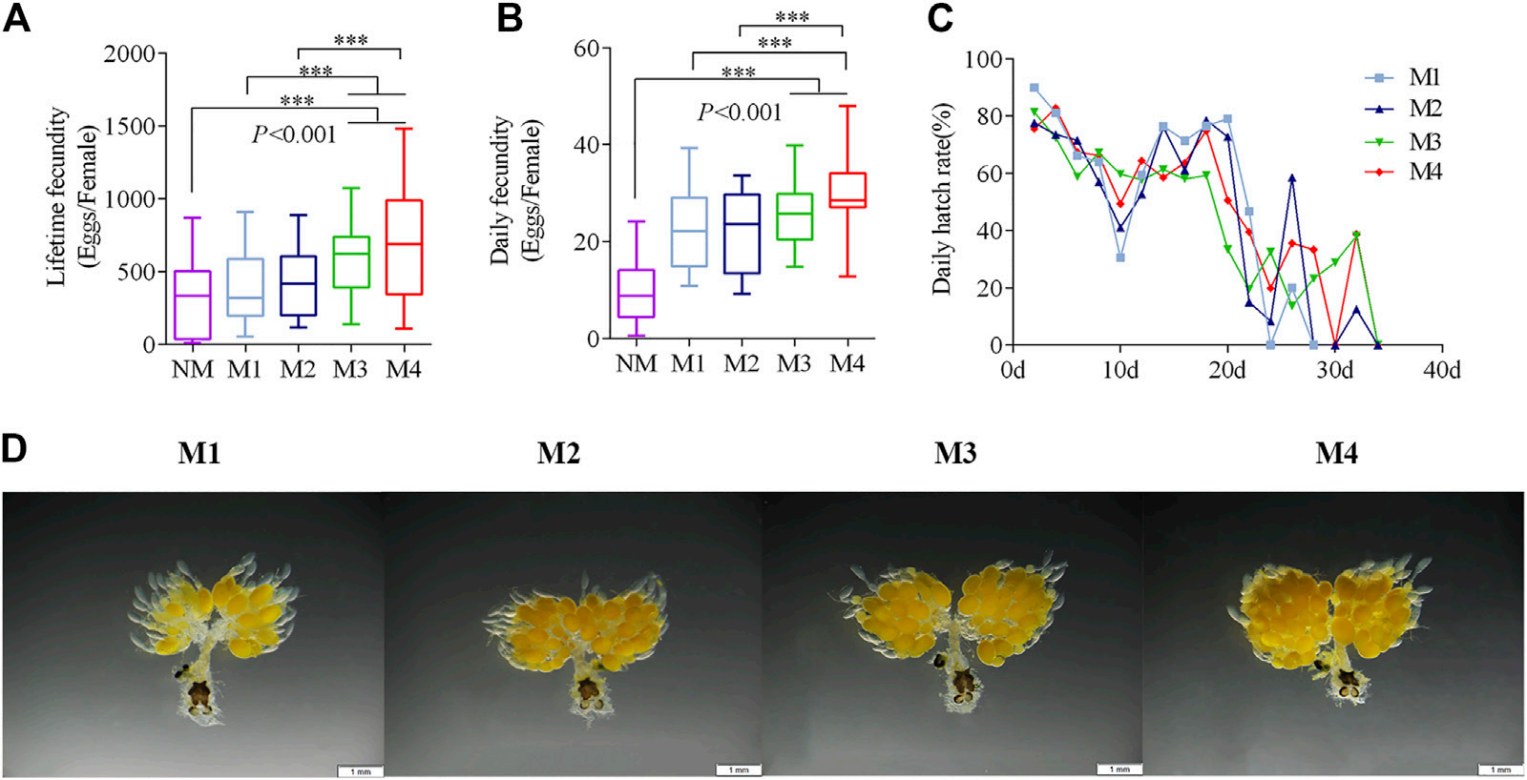
Effects of multiple matings on the fecundity and oogenesis. **(A–C)** Five-day-old Virgin males and females were paired and then separated when they had mated one (M1), two (M2), three (M3), and four (M4) times. Females that did Non-mating females (NM) acted as a control. (***p < 0.001, one-way ANOVA with an LSD test). **(D)** Oogenesis in *O. communa* is shown after different numbers of mating events at day 5.

The daily fecundity of the females significantly increased with the number of mating events (Figure 1A). Mating three and four times had significantly higher lifetime fecundity than females who were mating once and twice times (Figure 1B). The average lifetime fecundity of females that mating four times was 681.8 eggs on average (F = 7.63, *p* < 0.001), which was 1.7-fold higher than that of those mated only once. The hatching rate of different mating events was approximately the same (Figure 1C).

The ovaries began to develop 1 day after emergence. Eggs formed at 3–5 days and the ovariole was covered with closely arranged eggs. Subsequently, the ovaries entered the egg-maturation period. However, ovarian oogenesis gradually decreased if mating had not occurred by day 10 (Supplementary Figure S2). Mating frequency promoted egg production, which was most notable on the fifth day of the mating treatment (Figure 1D).

### Identification and sequence analysis of *OcCpb* and *OcCa*

Based on the transcriptomic data, we identified and cloned *Cpb* and *Ca* from *O. communa* (GenBank accession number: OQ134163 and OQ148164). The open reading frames for *OcCpb* and *OcCa* encoded 359 and 292 amino acid sequences, respectively Supplementary Figures S3, S4). Their molecular weights were 40.13 and 32.98 kDa and their isoelectric points were 8.69 and 6.17, respectively. *OcCpb* contained a zinc-binding region (KAVWIDGGIHAREWISPAVVTYI), characteristic of the zinc-dependent carboxypeptidase (Supplementary Figure S3). The motif SEHTIENYRFPLEMHLV was found to be highly conserved in *OcCa* (Supplementary Figure S4). Multiple sequence alignment showed that *Cpb* and *Ca* were highly conserved in *O. communa* and other Coleoptera species (Supplementary Figures S5, S6).

### Expression patterns of *OcCpb* and *OcCa*

**FIGURE 2:**
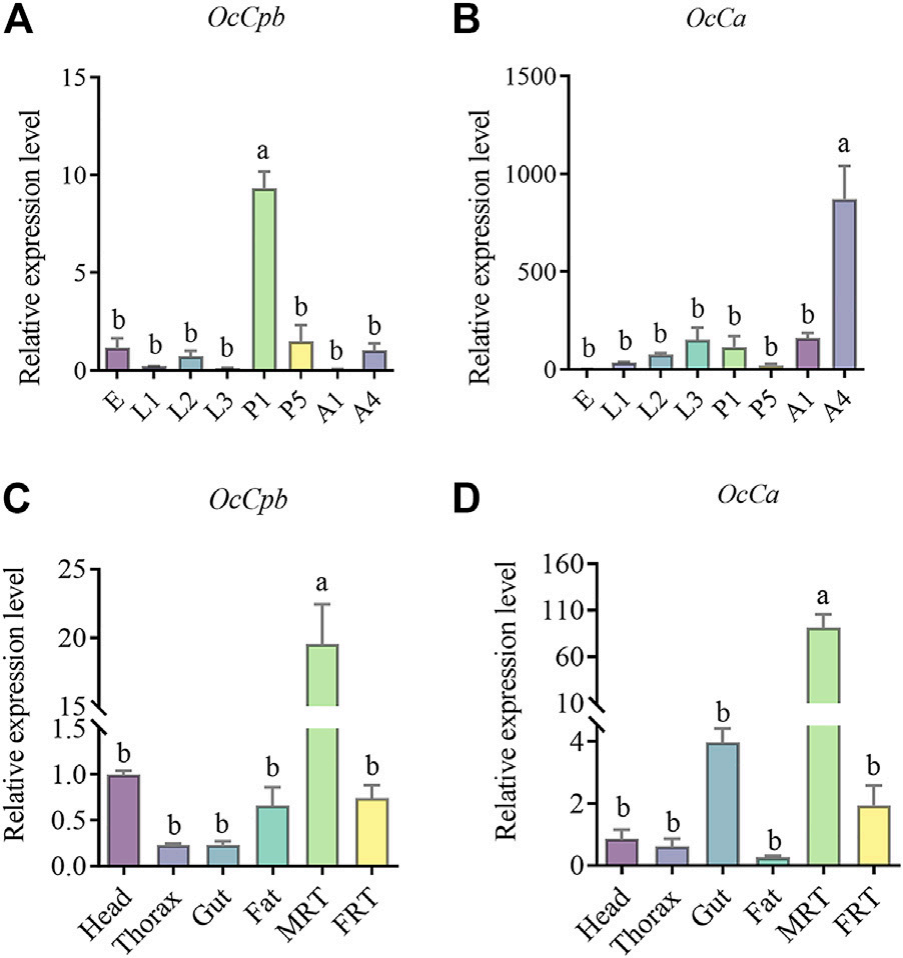
Relative expression levels of *OcCpb* and *OcCa* at different developmental stages and in different tissues. **(A,B)** Egg (E), first instar larva1 (L1), second instar larva (L2), third instar larva (L3), 1-day-old pupa (P1), 5-day-old pupa (P5), 1-day-old male adult (A1), 4-day-old male adult (A4). All expression fold changes are related to the egg. **(C,D)** Results are for the head, thorax, gut, fat, male reproductive tract (MRT), and female reproductive tract (FRT). All expression fold changes are related to head expression. The mRNA levels of *RPL4* were used as an internal standard. Different letters above the bars indicate significant differences at *p* < 0.05 level using the LSD test. (*n* = 3, mean ± SEM).

The *OcCpb* and *OcCa* expression levels showed notable developmental stage-specificity. Compared to the egg stage, *OcCpb* was significantly upregulated by 9.33-fold at the 1-day pupal stage (Figure 2A). Furthermore, *OcCa* expression levels in 4-day male adults were 871.37-fold higher than those at the egg stage (Figure 2B).

Gene expression was quantified in adult tissues and *OcCpb* and *OcCa* mRNA expression levels showed clear tissue specificity. Compared to that in the FRT, *OcCpb* and *OcCa* mRNA expression levels were significantly higher in the MRT (*p* < 0.01), where they had been upregulated by 26.20-fold and 85.02-fold, respectively (Figures 2C, D). The *OcCpb* and *OcCa* genes were virtually unexpressed in the thorax, gut, or fat of males. These results suggest that *OcCpb* and *OcCa* are putative seminal fluid protein genes in *O. communa* males.

### Expression analysis of *OcCpb* and *OcCa* the bursa copulatrix of females that had experienced different numbers of mating events

**FIGURE 3:**
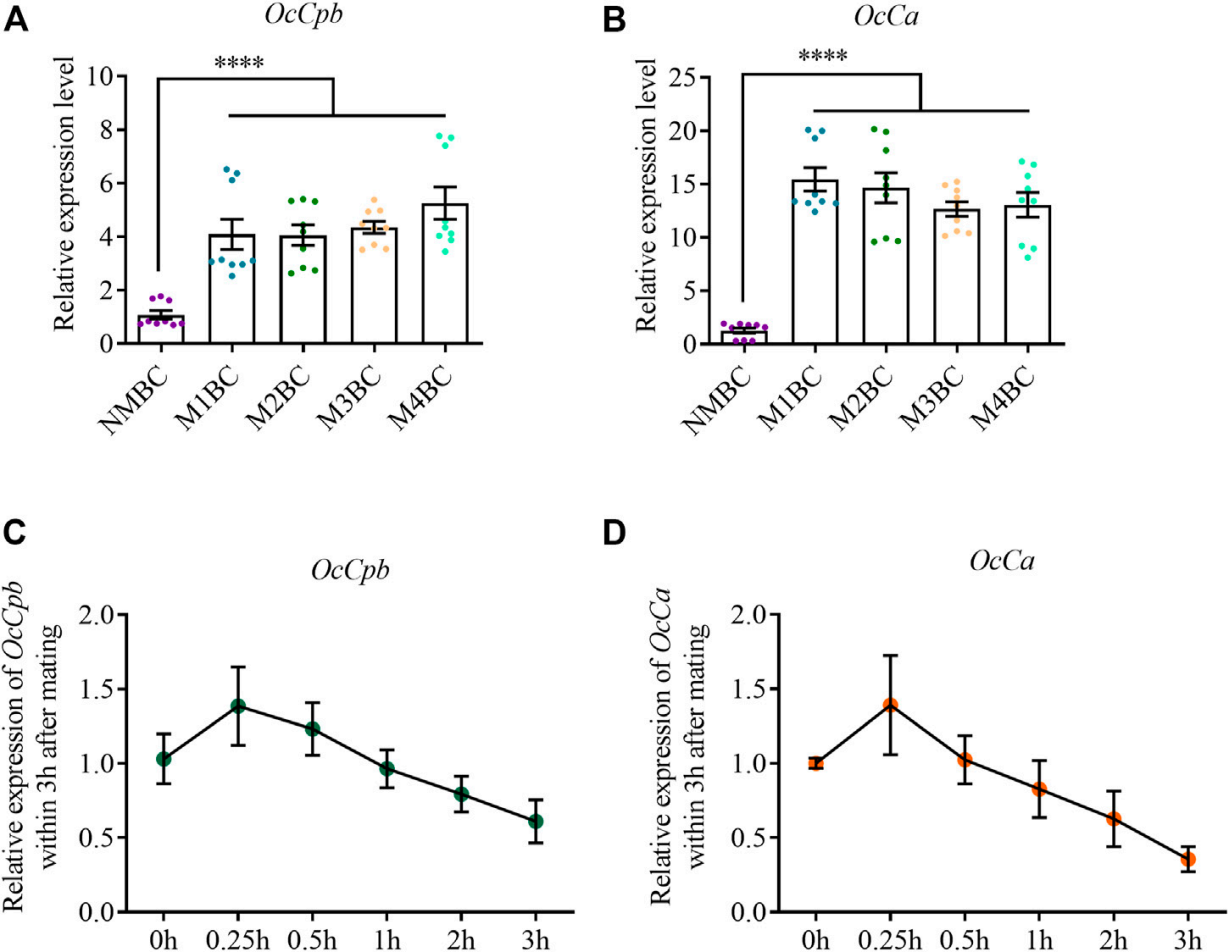
Expression analysis of *OcCpb* and *OcCa* in bursa copulatrix after different mating events. **(A,B)** Relative expression levels of *OcCpb* and *OcCa* in the bursa copulatrix. Non-mating bursa copulatrix (NMBC), mating once time bursa copulatrix (M1BC), mating twice times bursa copulatrix (M2BC), M3BC, M4BC and so on. All expression fold changes are related to NMBC. **(C,D)** Relative expression levels of *OcCpb* and *OcCa* in the bursa copulatrix within 3 h after mating. Virgin females and males (5-day-old) were paired, and then separated after 30 min. Bursa copulatrix was dissected right after separation at 0, 0.25, 0.5, 1, 2, and 3 h. All expression fold changes are related to 0 h (mean ± SEM, ****p < 0.0001, one-way ANOVA with LSD test).

*OcCpb* expression was upregulated after mating compared to that in the virgin bursa copulatrix. Its expression was highest after mating four times compared to that in NM females and was upregulated 7.05-fold (Figure 3A). Mating also increased *OcCa* expression in the bursa copulatrix. The highest expression was observed after mating once, which was 15.44-fold higher than that in NM females (Figure 3B). However, there was no significant difference in *OcCpb* and *OcCa* expression levels between the NM and post mating MRTs (Supplementary Figure S7).

We detected the expression of *OcCpb* and *OcCa* in the bursa copulatrix containing a spermatophore (Supplementary Figure S1). *OcCpb* expression was upregulated between 0 and 15 min, but was gradually downregulated after 15 min in the bursa copulatrix. It was downregulated by 38.9% up to 3 h after mating compared to that just after mating (Figure 3C). The *OcCa* expression changes in the mating-bursa copulatrix up to 3 h after mating were similar to *OcCpb*. The *OcCa* expression level was downregulated by 64.3% (Figure 3D).

### *OcCpb* and *OcCa* knockdown effects on reproduction

**FIGURE 4:**
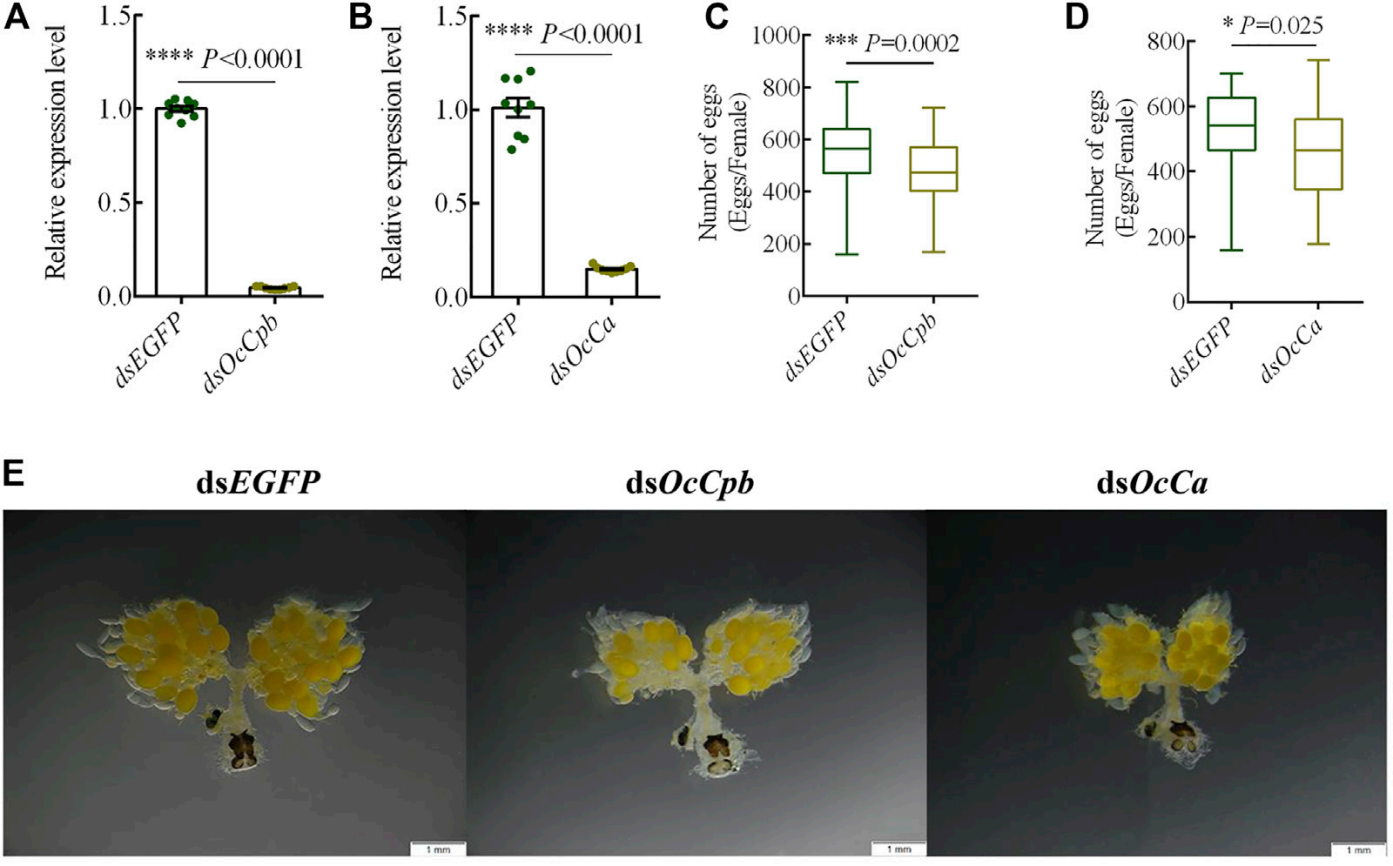
*OcCpb* and *OcCa* knockdown effects on male reproduction. **(A,B)** *OcCpb* and *OcCa* knockdown efficiencies in adults, respectively. The dsRNA was injected into newly emerged adults and the relative mRNA levels were measured using qPCR at 3 days post injection. Expression levels of the respective genes in control insects (injected *dsEGFP*) were set to 1 (*n* = 3; mean ± SEM). **(C,D)** Box plots show a significant difference in egg numbers between the dsRNA-treated groups and the controls. The RNAi males were mated with normal untreated females of the same age and then separated after mating for the first time. Females were reared alone. Number of eggs laid was determined at 15 days. (*p < 0.05, ***p < 0.001, ****p < 0.0001, one-way ANOVA with LSD test). **(E)** *OcCpb* and *OcCa* knockdown affected oogenesis. At 15 days after mating, the FRT was dissected and observed using stereo fluorescence microscopy.

To determine the function of *OcCpb* and *OcCa*, we injected dsRNA into males to knock down the expression of mRNA. This resulted in 90% and 85% reductions in *OcCpb* and *OcCa* expression levels in RNAi males, respectively (Figures 4A, B). The RNAi males were mated with untreated virgin females and the number of eggs produced by the females was recorded. The results showed that ovipositioning was significantly lower than that of the control after mRNA expression by *OcCpb* and *OcCa* had been disturbed. The number of eggs laid by the ds*OcCpb* group over 15 days was 474.1, whereas it was 542.5 for the control ds*EGFP* (F = 12.72, *p* < 0.001), which was a decrease in egg laying of 12.61% (Figure 4C). The number of eggs laid by the ds*OcCa* group over 15 days was 456.9, whereas it was 527.8 for the control ds*EGFP* (F = 5.21, *p* < 0.025), which was a 13.43% decrease in egg laying compared to that of the control (Figure 4D).

Moreover, the stimulation of mating behavior during ovarian oogenesis decreased. The ovaries of the treatment groups produced significantly fewer eggs compared to that produced by the control group. In addition, yolk sedimentation and the degree of ovarian tube loosening were greater in the control group than in the treatment groups (Figure 4E).

## Discussion

Mature females exhibited physiological ovulation behavior without mating, which provides a physiological basis for the strong reproductive ability of *O. communa*. Our data demonstrated that multiple matings promotes oogenesis, which supports the hypothesis that multiple matings brings direct benefits to females (Arnqvist and Nilsson, 2000; Caspers et al., 2014). This finding is also consistent with the results for multiple matings in other insects, such as *Colaphellus bozoringi* (Liu et al., 2013), *Galerucella birmanica* (Wang et al., 2018) and *Chrysochus asclepiadeus* (Schwartz and Peterson, 2006), where fecundity almost doubled on average when the organisms were allowed to mate repeatedly compared to that in individuals that mated once. Female lifetime fecundity generally increases with mating frequency, and these increases are considered to be due to the sufficient amount of sperm or SFPs in the male ejaculate for fertilization (Arnqvist and Nilsson, 2000; Avila et al., 2011). After females are stimulated by SFPs, ovarian oogenesis is accelerated, and the number of eggs laid by females increases (Wolfner, 2002; Tseng et al., 2007). SFPs are necessary for the efficient utilization of stored sperm, with the few sperm stored in the absence of SFPs not used to fertilize eggs in *D. melanogaster* females (Xue and Noll, 2000). The reproductive success of both sexes is adversely affected when SFPs are absent from the ejaculate (Avila et al., 2011). Therefore, *O. communa* adults have a high reproductive capacity, which is related to both the physiological foundation of females and the regulation of SFPs in males.

Many SFPs have been identified in insects, and most SFPs identification studies have examined RNA or proteins found in the tissues of the MRT (Avila et al., 2011). The expression patterns of genes encoding 13 of the 14 proteins identified by South et al. (South et al., 2011) were highly male-biased, with undetectable transcript levels in the female tissues. Furthermore, genes encoding 15 SFPs were highly expressed in *Callosobruchus maculatus* male abdomens, but were only negligibly expressed in females. In our study, the expression patterns of *OcCpb* and *OcCa* were also highly male-biased (Figure 2). These results were similar to the expression profiles for SFP genes in *T. castaneum* and *C. maculatus*. Thus, combined with the findings reported by Gao et al. (2020) reported, two lines of evidence suggest that *OcCpb* and *OcCa* with male-specific gene expression levels represent putative SFPs: 1) they were identified in mating bursa copulatrix but were not detected in the NM bursa copulatrix and 2) the qPCR revealed low expression levels in females.

Cpb and Ca have been identified as SFPs in *O. communa*, but their reproductive functions in insects have rarely been reported. Previous studies have reported that Cpb plays an important role in the degradation of dietary proteins in the insects intestines (Bown and Gatehouse, 2004). When the activity of Cpb was inhibited, the development of parasites in the mosquito midgut was blocked in *Anopheles gambiae* (Lavazec et al., 2007). In this study, we determined that *OcCpb* and *OcCa* mediates the female fertility (Figure 4). A non-specific carboxylesterase with proteolytic activity is present in the secretions compartmentalized closer to the *D. melanogaster* male genital opening in the ejaculatory duct and is transported almost immediately after mating begins. (Meikle et al., 1990). Zymogen Cpb, synthesized and secreted in the male reproductive organs, was reported to be catabolically activated post mating and in the female reproductive organs to initiate the molecular pathway of the fertilized egg in the silkworm (Sakakura et al., 2022). It appears that *OcCpb* influences fertilization egg formation by the same mechanism and involved in the regulation of reproduction in insects. Ca catalyzes the reversible hydration of carbon dioxide and bicarbonate anion. It was confirmed that HCO_3_^−^ involved in fertilized egg formation by inducing intracellular alkalinization and by directly accelerating sperm movement (Wennemuth et al., 2003). In recent years, research on the relationship between Ca and fertilization has mainly been conducted in higher animals. There are 14 subtypes of this protein, which are mainly expressed in the testes and epididymis of rats, rabbits, cows, and humans (Ekstedt et al., 2003). Human sperm and egg fuse to generate a new individual strongly depends on Ca activity (José et al., 2015). It is also important to mention that Ca can create both physical and functional metabolons with a variety of different anion exchangers, which further complicates the role of Ca in fertilization (Ali Akbar et al., 1998; Del Prete et al., 2014). Hence, *OcCa* may indirectly affect fertilization through bicarbonate ions. To the best of our knowledge, this study is the first study to identify *Cpb* and *Ca* as SFP genes that play a regulatory role in insects’ reproduction.

The mating bursa copulatrix contains a newly transferred spermatophore. It also includes sperm encapsulated by SFPs, which is similar to those in ground crickets (*Allonemobius socius*) (Marshall et al., 2009)*.* The spermatophore in the bursa copulatrix is an active substance that acts as a signaling factor and stimulates hormonal effects in females (Wolfner, 2002; Fiumera et al., 2007; Mueller et al., 2008). SFPs can induce gene expression post mating (McGraw et al., 2008; Amaro et al., 2021). Acp levels continue to increase within the *D. melanogaster* female genital tract throughout mating (Lung and Wolfner, 1999). Similarly, the expression of *OcCpb* and *OcCa* upregulates in the mating bursa copulatrix. The phenomenon that *OcCpb* and *OcCa* upregulation in the bursa copulatrix with different mating events does not show a linear increase in mating frequency, we believe it is most likely due to the inconsistency of each mating time interval. Moreover, new SFPs were transferred to females and previous SFPs were consumed when there were two mating events. Some SFPs transferred from the *D. melanogaster* male enter the female’s circulatory system whereas others are confined within the reproductive tract (Lung and Wolfner, 1999; Neubaum and Wolfner, 1999). Given this, we hypothesized that the SFP genes related to multiple mating to promote egg production should be downregulated due to the SFPs consumption by females during the interval between two mating. Interestingly, the expression of *OcCpb* and *OcCa* in mating bursa copulatrix was lower in the 3 h than at the 15 min, which were consistent with our expectations. Similar trends are seen with Acp26Aa, Acp26Ab, and Acp62F (Monsma et al., 1990; Lung and Wolfner, 1999). Moreover, levels of two *D. melanogaster* SFPs (ovulin and sex peptide) decline in the mated female with time since mating (Sirot et al., 2009). It may be the result from the decrease or termination of SFP entry into the female genital tract or hemolymph in post mating along with removal of SFP from the general circulation as a result of metabolism or receptor binding at their target tissues (Lung and Wolfner, 1999). Downregulation of SFP genes occur with each mating events, which continuously stimulates the female to respond. It seems to imply that *OcCpb* and *OcCa* have a cumulative effect and play a reproductive regulatory role in multiple matings.

In summary, we have demonstrated that multiple matings promotes oogenesis and identified that *Cpb* and *Ca* are putative seminal fluid protein genes in *O. communa*. Although these genes in the bursa copulatrix did not show a linear increase with mating frequency, they were upregulated during different mating events and gradually downregulated after finishing copulation. Additionally, *OcCpb* and *OcCa* mediate oogenesis and female fertility, which provided insights into the relevance of them involvement in multiple matings. Further studies are required to identify the various roles played by *OcCpb* and *OcCa* in regulating male reproduction, especially regarding SFP synthesis and secretion.

## Data Availability

The original contributions presented in the study are included in the article/Supplementary Material, further inquiries can be directed to the corresponding author.

## References

[B1] Aigaki T. Osanai M. Kasuga H. (1988). Arginine carboxypeptidase activity in the male reproductive glands of the silkworm, *Bombyx mori* . Bombyx Mori. Insect Biochem. 18, 295–298. 10.1016/0020-1790(88)90094-7

[B2] Alcock J. Barrows E. M. Gordh G. Hubbard L. J. Kirkendall L. Pyle D. W. (1978). The ecology and evolution of male reproductive behaviour in the bees and wasps. Zoological J. Linn. Soc. 64, 293–326. 10.1111/j.1096-3642.1978.tb01075.x

[B3] Ali Akbar S. Nicolaides K. H. Brown P. R. (1998). Carbonic anhydrase isoenzymes CAI and CAII in semen, decidua, chorionic villi and various fetal tissues. Early Hum. Dev. 51, 205–211. 10.1016/S0378-3782(97)00119-9 9692790

[B4] Amaro I. A. Ahmed-Braimah Y. H. League G. P. Pitcher S. A. Avila F. W. Cruz P. C. (2021). Seminal fluid proteins induce transcriptome changes in the *Aedes aegypti* female lower reproductive tract. BMC Genomics 22, 896. 10.1186/s12864-021-08201-0 34906087 PMC8672594

[B5] Arnqvist G. Nilsson T. (2000). The evolution of polyandry: Multiple mating and female fitness in insects. Anim. Behav. 60, 145–164. 10.1006/anbe.2000.1446 10973716

[B6] Avila F. W. Sirot L. K. LaFlamme B. A. Rubinstein C. D. Wolfner M. F. (2011). Insect seminal fluid proteins: Identification and function. Annu. Rev. Entomol. 56, 21–40. 10.1146/annurev-ento-120709-144823 20868282 PMC3925971

[B7] Barrett A. J. Woessner J. F. Rawlings N. D. (2012). Handbook of proteolytic enzymes, Vol. 1. Amsterdam, Netherlands: Elsevier.

[B8] Bloch Qazi M. C. Heifetz Y. Wolfner M. F. (2003). The developments between gametogenesis and fertilization: Ovulation and female sperm storage in *Drosophila melanogaster* . Dev. Biol. 256, 195–211. 10.1016/S0012-1606(02)00125-2 12679097

[B9] Bown D. P. Gatehouse J. A. (2004). Characterization of a digestive carboxypeptidase from the insect pest corn earworm (*Helicoverpa armigera*) with novel specificity towards C-terminal glutamate residues. Eur. J. Biochem. 271, 2000–2011. 10.1111/j.1432-1033.2004.04113.x 15128309

[B10] Caesar S. Forsman A. (2009). Do polyandrous pygmy grasshopper females obtain fitness benefits for their offspring? Behav. Ecol. 20, 354–361. 10.1093/beheco/arn153

[B11] Caspers B. A. Krause E. T. Hendrix R. Kopp M. Rupp O. Rosentreter K. (2014). The more the better - polyandry and genetic similarity are positively linked to reproductive success in a natural population of terrestrial salamanders *(Salamandra salamandra)* . Mol. Ecol. 23, 239–250. 10.1111/mec.12577 24168518

[B12] Del Prete S. Vullo D. Fisher G. M. Andrews K. T. Poulsen S.-A. Capasso C. (2014). Discovery of a new family of carbonic anhydrases in the malaria pathogen plasmodium falciparum —the η-carbonic anhydrases. Bioorg. Med. Chem. Lett. 24, 4389–4396. 10.1016/j.bmcl.2014.08.015 25168745

[B13] Eberhard W. (1996). Female control: Sexual selection by cryptic female choice. United States: Princeton University Press.

[B14] Ekstedt E. Holm L. Ridderstråle Y. (2003). Carbonic anhydrase in mouse testis and epididymis; transfer of isozyme iv to spermatozoa during passage. Histochem J. 35, 167–173. 10.1023/B:HIJO.0000023387.02793.af 15328921

[B15] Fiumera A. C. Dumont B. L. Clark A. G. (2007). Associations between sperm competition and natural variation in male reproductive genes on the third chromosome of *Drosophila melanogaster* . Genetics 176, 1245–1260. 10.1534/genetics.106.064915 17435238 PMC1894588

[B16] Futuyma D. J. McCafferty S. S. (1990). Phylogeny and the evolution of host plant associations in the leaf beetle *Genus Ophraella* (Coleoptera, Chrysomelidae). Evolution 44, 1885–1913. 10.1111/j.1558-5646.1990.tb04298.x 28564433

[B17] Gao X. Y. Tian Z. Y. Zhang Y. Chen G. M. Ma C. Tian Z. Q. (2020). Transcriptome analysis of *Ophraella communa* male reproductive tract in indirect response to elevated CO_2_ and heat wave. Front. Physiol. 11, 417. 10.3389/fphys.2020.00417 32431624 PMC7215069

[B18] Guo J. Y. Zhou Z. S. Zheng X. W. Chen H. S. Wan F. H. Luo Y. H. (2011). Control efficiency of leaf beetle, *Ophraella communa*, on the invasive common ragweed, *Ambrosia artemisiifolia*, at different growing stages. Biocontrol Sci. Technol. 21, 1049–1063. 10.1080/09583157.2011.603823

[B19] Hu Y. Meng L. (2007). Potential impact of alien herbivorous insect *Ophraella communa* (Coleoptera: Chrysomelidae) on non-target plants in mainland China. Chin. J. Ecol. 26, 56–60.

[B20] Immarigeon C. Frei Y. Delbare S. Y. N. Gligorov D. Machado Almeida P. Grey J. (2021). Identification of a micropeptide and multiple secondary cell genes that modulate *Drosophila* male reproductive success. Proc. Natl. Acad. Sci. U.S.A. 118, e2001897118. 10.1073/pnas.2001897118 33876742 PMC8053986

[B21] Inaba K. Dréanno C. Cosson J. (2003). Control of flatfish sperm motility by CO_2_ and carbonic anhydrase: Carbonic anhydrase and sperm motility. Cell Motil. Cytoskelet. 55, 174–187. 10.1002/cm.10119 12789662

[B22] Jennions M. D. Petrie M. (2007). Why do females mate multiply? A review of the genetic benefits. Biol. Rev. 75, 21–64. 10.1017/s0006323199005423 10740892

[B23] José O. Torres-Rodríguez P. Forero-Quintero L. S. Chávez J. C. De la Vega-Beltrán J. L. Carta F. (2015). Carbonic anhydrases and their functional differences in human and mouse sperm physiology. Biochem. Biophysical Res. Commun. 468, 713–718. 10.1016/j.bbrc.2015.11.021 26551457

[B24] Koene J. M. Sloot W. Montagne-Wajer K. Cummins S. F. Degnan B. M. Smith J. S. (2010). Male accessory gland protein reduces egg laying in a simultaneous hermaphrodite. PLoS ONE 5, e10117. 10.1371/journal.pone.0010117 20404934 PMC2853560

[B25] Lavazec C. Boudin C. Lacroix R. Bonnet S. Diop A. Thiberge S. (2007). Carboxypeptidases B of *Anopheles gambiae* as targets for a *Plasmodium falciparum* transmission-blocking vaccine. Infect. Immun. 75, 1635–1642. 10.1128/iai.00864-06 17283100 PMC1865713

[B26] Lewis S. M. Jutkiewicz E. (1998). Sperm precedence and sperm storage in multiply mated red flour beetles. Behav. Ecol. Sociobiol. 43, 365–369. 10.1007/s002650050503

[B27] Liu X. P. He H. M. Xue F. S. (2013). The effect of mating frequency and mating pattern on female reproductive fitness in cabbage beetle, *Colaphellus bowringi* . Entomol. Exp. Appl. 146, 379–385. 10.1111/eea.12037

[B28] Lung O. Wolfner M. F. (1999). Drosophila seminal fluid proteins enter the circulatory system of the mated female fly by crossing the posterior vaginal wall. Insect Biochem. Mol. Biol. 29, 1043–1052. 10.1016/S0965-1748(99)00078-8 10612039

[B29] Lung O. Wolfner M. F. (2001). Identification and characterization of the major *Drosophila melanogaster* mating plug protein. Insect Biochem. Mol. Biol. 31, 543–551. 10.1016/S0965-1748(00)00154-5 11267893

[B30] Ma C. Cui S. W. Bai Q. Tian Z. Y. Zhang Y. Chen G. M. (2020). Olfactory co‐receptor is involved in host recognition and oviposition in *Ophraella communa* (Coleoptera: Chrysomelidae). Insect Mol. Biol. 29, 381–390. 10.1111/imb.12643 32291884

[B31] Mann T. Mann C. L. Dixon R. L. (1982). Passage of chemicals into human and animal semen: Mechanisms and significance. CRC Crit. Rev. Toxicol. 11, 1–14. 10.3109/10408448209089846 6761065

[B32] Marshall J. L. Huestis D. L. Hiromasa Y. Wheeler S. Oppert C. Marshall S. A. (2009). Identification, RNAi knockdown, and functional analysis of an ejaculate protein that mediates a postmating, prezygotic phenotype in a Cricket. PLoS ONE 4, e7537. 10.1371/journal.pone.0007537 19851502 PMC2761614

[B34] McGraw L. A. Clark A. G. Wolfner M. F. (2008). Post-mating gene expression profiles of female *Drosophila melanogaster* in response to time and to four male accessory gland proteins. Genetics 179, 1395–1408. 10.1534/genetics.108.086934 18562649 PMC2475742

[B35] Meikle D. B. Sheehan K. B. Phillis D. M. Richmond R. C. (1990). Localization and longevity of seminal-fluid esterase 6 in mated female *Drosophila melanogaster* . J. Insect Physiology 36, 93–101. 10.1016/0022-1910(90)90179-J

[B36] Meldrum N. U. Roughton F. J. W. (1933). Carbonic anhydrase. Its preparation and properties. J. Physiology 80, 113–142. 10.1113/jphysiol.1933.sp003077 PMC1394121 16994489

[B37] Meng L. Li B. (2005). Advances on biology and host specificity of the newly introduced beetle, *Ophraella communa* Lesage (Coleoptera:Chrysomelidae), attacking *Ambrosia artemisiifolia* (Compositae) in continent of China. Chin. J. Biol. Control 21, 65–69.

[B38] Mirjafari P. Asghari K. Mahinpey N. (2007). Investigating the application of enzyme carbonic anhydrase for CO_2_ sequestration purposes. Ind. Eng. Chem. Res. 46, 921–926. 10.1021/ie060287u

[B39] Monsma S. A. Harada H. A. Wolfner M. F. (1990). Synthesis of two *Drosophila* male accessory gland proteins and their fate after transfer to the female during mating. Dev. Biol. 142, 465–475. 10.1016/0012-1606(90)90368-S 2257979

[B40] Mueller J. L. Linklater J. R. Ravi Ram K. Chapman T. Wolfner M. F. (2008). Targeted gene deletion and phenotypic analysis of the *Drosophila melanogaster* seminal fluid protease inhibitor Acp62F. Genetics 178, 1605–1614. 10.1534/genetics.107.083766 18245332 PMC2278106

[B41] Neubaum D. M. Wolfner M. F. (1999). Mated *Drosophila melanogaster* females require a seminal fluid protein, Acp36DE, to store sperm efficiently. Genetics 153, 845–857. 10.1093/genetics/153.2.845 10511562 PMC1460804

[B42] Newcomer S. D. Zeh J. A. Zeh D. W. (1999). Genetic benefits enhance the reproductive success of polyandrous females. Proc. Natl. Acad. Sci. U.S.A. 96, 10236–10241. 10.1073/pnas.96.18.10236 10468592 PMC17872

[B43] Patlar B. Civetta A. (2022). Seminal fluid gene expression and reproductive fitness in *Drosophila melanogaster* . BMC Ecol. Evo 22, 20. 10.1186/s12862-022-01975-1 PMC8867848 35196983

[B33] Mason P. G. Huber J. T. (Editors) (2002). Biological control programmes in Canada, 1981-2000 (Oxon, UK ; New York: CABI Pub).

[B44] Poiani A. (2006). Complexity of seminal fluid: A review. Behav. Ecol. Sociobiol. 60, 289–310. 10.1007/s00265-006-0178-0

[B45] Pondeville E. Maria A. Jacques J.-C. Bourgouin C. Dauphin-Villemant C. (2008). *Anopheles gambiae* males produce and transfer the vitellogenic steroid hormone 20-hydroxyecdysone to females during mating. Proc. Natl. Acad. Sci. U.S.A. 105, 19631–19636. 10.1073/pnas.0809264105 19060216 PMC2604965

[B46] Sakakura M. Takata Y. Kimura C. Matsuda S. Takamura T. Nagaoka S. (2022). Limited proteolysis by a prostatic endopeptidase, the sperm-activating factor initiatorin, regulates the activation of pro-carboxypeptidase B in the seminal fluid of the silkworm, *Bombyx mori* . Insect Biochem. Mol. Biol. 148, 103819. 10.1016/j.ibmb.2022.103819 35963292

[B47] Schwartz S. K. Peterson M. A. (2006). Strong material benefits and no longevity costs of multiple mating in an extremely polyandrous leaf beetle, *Chrysochus cobaltinus* (Coleoptera: Chrysomelidae). Behav. Ecol. 17, 1004–1010. 10.1093/beheco/arl033

[B48] Simmons L. W. (2019). Sperm competition and its evolutionary consequences in the insects. United States: Princeton University Press.

[B49] Sirot L. K. Buehner N. A. Fiumera A. C. Wolfner M. F. (2009). Seminal fluid protein depletion and replenishment in the fruit fly, *Drosophila melanogaster*: An ELISA-based method for tracking individual ejaculates. Behav. Ecol. Sociobiol. 63, 1505–1513. 10.1007/s00265-009-0806-6 24733957 PMC3984576

[B50] Smith K. S. Jakubzick C. Whittam T. S. Ferry J. G. (1999). Carbonic anhydrase is an ancient enzyme widespread in prokaryotes. Proc. Natl. Acad. Sci. U.S.A. 96, 15184–15189. 10.1073/pnas.96.26.15184 10611359 PMC24794

[B51] South A. Sirot L. K. Lewis S. M. (2011). Identification of predicted seminal fluid proteins in *Tribolium castaneum: Tribolium castaneum* seminal fluid proteins. Insect Mol. Biol. 20, 447–456. 10.1111/j.1365-2583.2011.01083.x 21689183

[B52] Tian Z. Q. Zhang Y. Ma C. Chen H. S. Guo J. Y. Zhou Z. S. (2020). Silencing the myosin regulatory light chain gene sqh reduces cold hardiness in *Ophraella communa* LeSage (Coleoptera: Chrysomelidae). Insects 11, 844. 10.3390/insects11120844 33260791 PMC7768443

[B53] Tseng H. F. Yang R. L. Lin C. Horng S. B. (2007). The function of multiple mating in oviposition and egg maturation in the seed beetle *Callosobruchus maculatus* . Physiol. Entomol. 32, 150–156. 10.1111/j.1365-3032.2007.00561.x

[B54] Wang L. Y. Meng M. Wang Y. M. (2018). Repeated mating with the same male increases female longevity and fecundity in a polyandrous leaf beetle *Galerucella birmanica* (Coleoptera: Chrysomelidae): Benefit of repeated mating in *G. birmanica* . Physiol. Entomol. 43, 100–107. 10.1111/phen.12233

[B55] Ward K. E. Landolt P. J. (1995). Influence of multiple matings on fecundity and longevity of female cabbage looper moths (Lepidoptera: Noctuidae). Ann. Entomological Soc. Am. 88, 768–772. 10.1093/aesa/88.6.768

[B56] Wennemuth G. Carlson A. E. Harper A. J. Babcock D. F. (2003). Bicarbonate actions on flagellar and Ca^2+^-channel responses: Initial events in sperm activation. Development 130, 1317–1326. 10.1242/dev.00353 12588848

[B57] Wolfner M. F. (2002). The gifts that keep on giving: Physiological functions and evolutionary dynamics of male seminal proteins in *Drosophila* . Heredity 88, 85–93. 10.1038/sj.hdy.6800017 11932766

[B58] Xu J. Baulding J. Palli S. R. (2013). Proteomics of *Tribolium castaneum* seminal fluid proteins: Identification of an angiotensin-converting enzyme as a key player in regulation of reproduction. J. Proteomics 78, 83–93. 10.1016/j.jprot.2012.11.011 23195916

[B59] Xu J. Wang Q. (2011). Seminal fluid reduces female longevity and stimulates egg production and sperm trigger oviposition in a moth. J. Insect Physiology 57, 385–390. 10.1016/j.jinsphys.2010.12.006 21172356

[B60] Xu X. Chen J. Du X. Yao L. S. Wang Y. Q. (2022). CRISPR/Cas9 mediated disruption of seminal fluid protein Sfp62 induces male sterility in *Bombyx mori* . Biology 11, 561. 10.3390/biology11040561 35453761 PMC9024854

[B61] Xue L. Noll M. (2000). *Drosophila* female sexual behavior induced by sterile males showing copulation complementation. Proc. Natl. Acad. Sci. U.S.A. 97, 3272–3275. 10.1073/pnas.060018897 10725377 PMC16228

[B62] Zheng X. W. Zhou Z. S. Guo J. Y. Wan F. H. Chen H. S. Wang J. G. (2011). Effect of initial densities on population expansion of *Ophraella communa* . J. Environ. Entomology 33, 128–130.

[B63] Zhou Z. S. Guo J. Y. Chen H. S. Wan F. H. (2010). Effects of temperature on survival, development, longevity, and fecundity of *Ophraella communa* (Coleoptera: Chrysomelidae), a potential biological control agent against *Ambrosia artemisiifolia* (asterales: Asteraceae). Environ. Entomol. 39, 1021–1027. 10.1603/EN09176 20550818

[B64] Zhou Z. S. Guo J. Y. Wan F. H. (2009). Biological control of Ambrosia artemisiifolia with Epibleme strenuana and *Ophraella communa* . Res. Biol. invasions China 2009, 253–258.

[B65] Zhou Z. S. Guo J. Y. Zheng X. W. Luo M. Chen H. S. Wan F. H. (2011). Reevaluation of biosecurity of *Ophraella communa* against sunflower (*Helianthus annuus*). Biocontrol Sci. Technol. 21, 1147–1160. 10.1080/09583157.2011.606559

[B66] Zhou Z. S. Rasmann S. Zheng H. Y. Watson A. Guo J. Y. Wang J. G. (2015). Mating frequency positively associates with fitness in *Ophraella communa* . Ecol. Entomol. 40, 292–298. 10.1111/een.12184

