# Supplementary material for: The carboxypeptidase B and carbonic anhydrase genes play a reproductive regulatory role during multiple matings in Ophraella communa
Source: Front Mol Biosci. 2023 May 17;10:1095645. doi: 10.3389/fmolb.2023.1095645 (PMC10229896; doi:10.3389/fmolb.2023.1095645)
Supplement: Supplementary file 1 [file Table1.DOCX]

**Supplementary material**


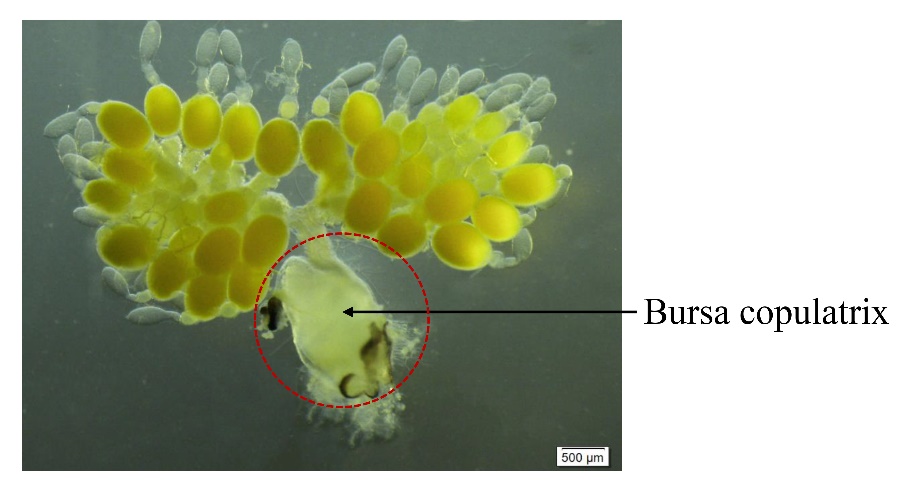


**Supplementary Figure S1.** The female reproductive tract (FRT) of *Ophraella communa* (ventral view).


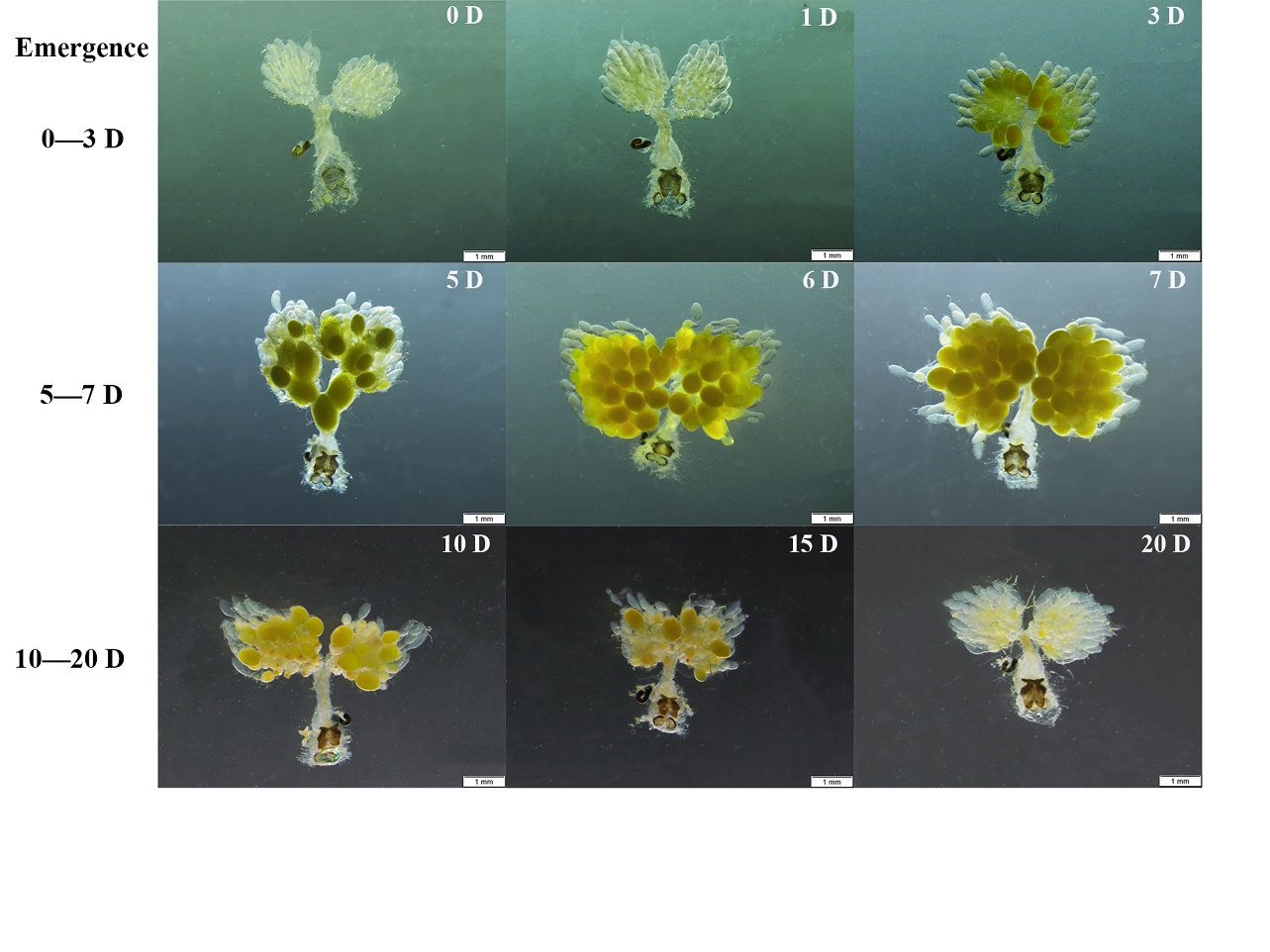


**Supplementary Figure S2.** Female reproductive tract of *Ophraella communa* in different developmental stages.


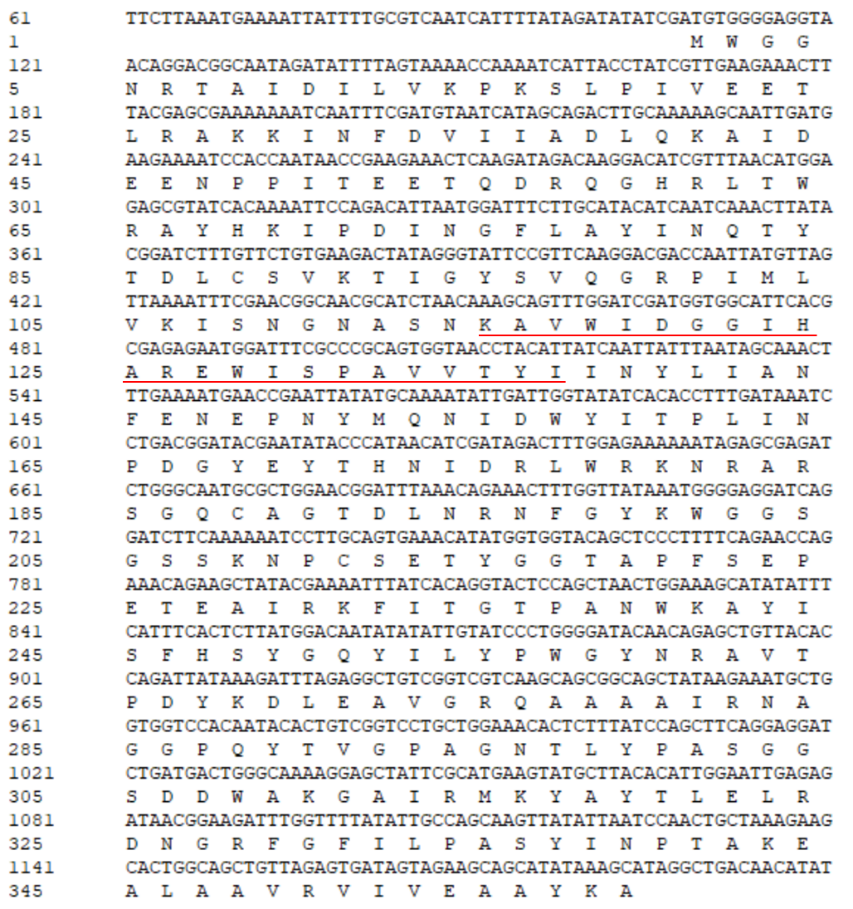


**Supplementary Figure S3.** Nucleotide and deduced amino acid sequences of *Cpb* cDNA from *O.communa*. The amino acid sequence derived from the cloned gene was analyzed, and the amino acid polypeptide chain contained a highly conserved zinc-binding region of KAVWIDGGIHAREWISPAVVTYI, which was represented by red lines.


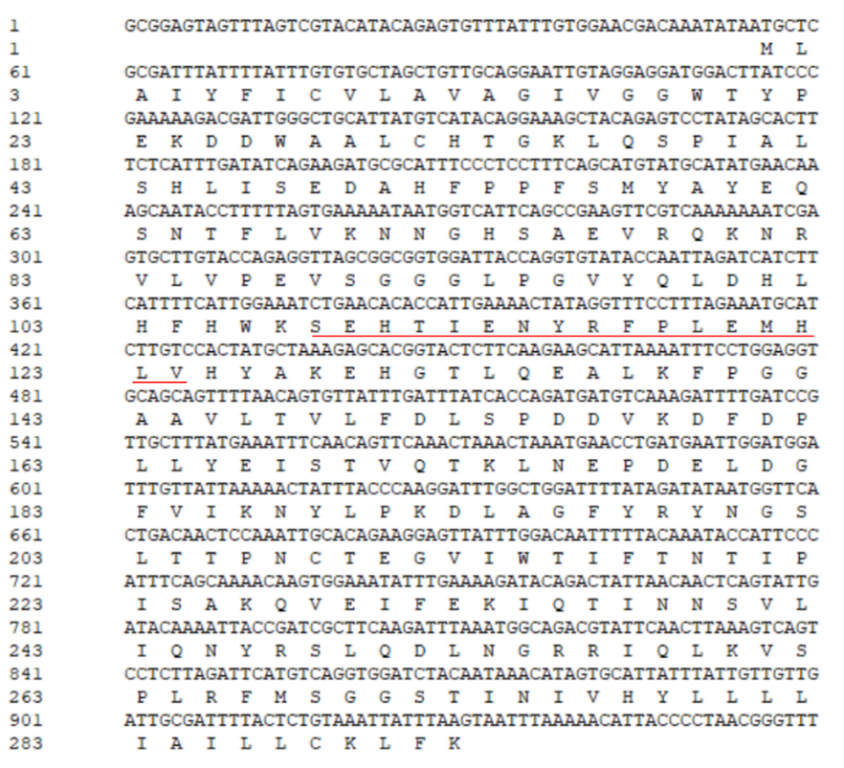


**Supplementary Figure S4.** Nucleotide and deduced amino acid sequences of *Ca* cDNA from *O.communa*. The amino acid sequence derived from the cloned gene was analyzed, and the amino acid polypeptide chain contained a highly conserved site of SEHTIENYRFPLEMHLV, which was represented by red lines.

**Supplementary Figure S5.** Multiple sequence alignment of *Cpb* from *O. communa* and other Coleopteran.

**Supplementary Figure S6.** Multiple sequence alignment of *Ca* from *O. communa* and other Coleopteran.


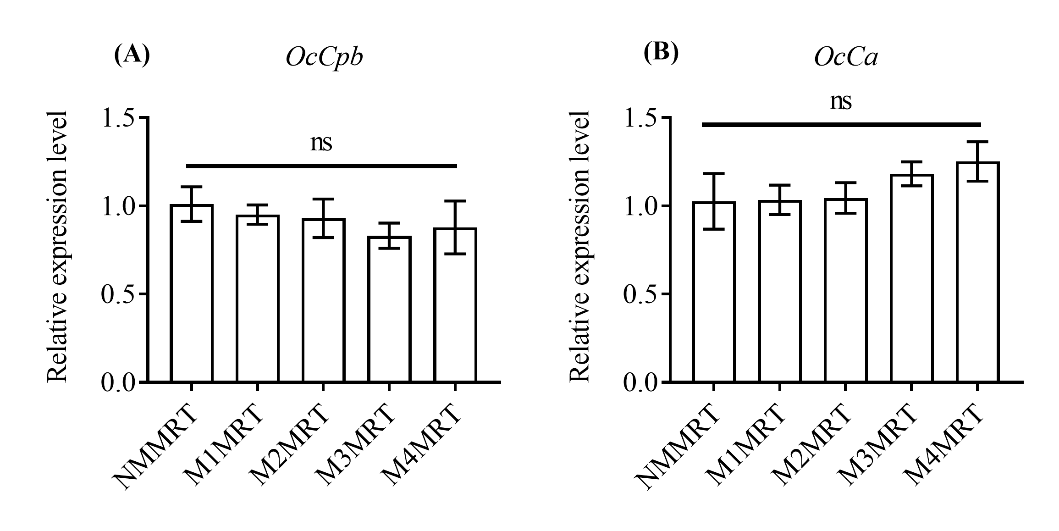


**Supplementary** **Figure S7.** Expression level of *OcCpb* and *OcCa* in the non-mating and post-mating male reproductive tract (MRT).

**Supplementary Table S1.** The primers used in this study.

| Gene name | Forward primer | Reverse primer |
| --- | --- | --- |
| **RT-PCR** |  |  |
| *OcCpb* | TGCCTCATTATGCCAATCT | GATATGTTGTCAGCCTATGC |
| *OcCa* | GCGGAGTAGTTTAGTCGTA | GCTTTGGTACAACTTTGGTA |
| **qRT-PCR** |  |  |
| *RPL4* | GTGTAGATATGGAGCGAATG | GTTAGCGAGCACTAGAATC |
| *OcCpb* | CAGGAGGATCTGATGACT | CTGCCAGTGCTTCTTTAG |
| *OcCa* | GGAATTGTAGGAGGATGG | GCTGAAAGGAGGGAAATG |
| **dsRNA** |  |  |
| ds*EGFP* | TGAGCAAGGGCGAGGAG | CGGCGGTCACGAACTCCAG |
| ds*OcCpb* | GCAACGCATCTAACAAAG | GGGAGCTGTACCACCATA |
| ds*OcCa* | TTGTAGGAGGATGGACTTA | AGTACCGTGCTCTTTAGC |
